# Depressive Symptomatology in Early Retirees Associated With Reason for Retirement—Results From the Population-Based LIFE-Adult-Study

**DOI:** 10.3389/fpsyt.2020.565442

**Published:** 2020-09-18

**Authors:** Andrea E. Zuelke, Susanne Roehr, Matthias L. Schroeter, A. Veronica Witte, Andreas Hinz, Heide Glaesmer, Christoph Engel, Cornelia Enzenbach, Silke Zachariae, Samira Zeynalova, Markus Loeffler, Arno Villringer, Steffi G. Riedel-Heller

**Affiliations:** ^1^ Institute of Social Medicine, Occupational Health and Public Health (ISAP), University of Leipzig, Leipzig, Germany; ^2^ Global Brain Health Institute (GBHI), Trinity College Dublin, Dublin, Ireland; ^3^ Department of Neurology, Max-Planck-Institute for Human Cognitive and Brain Sciences, Leipzig, Germany; ^4^ University Hospital Leipzig, Clinic for Cognitive Neurology, Leipzig, Germany; ^5^ Department of Medical Psychology and Medical Sociology, University of Leipzig, Leipzig, Germany; ^6^ Institute for Medical Informatics, Statistics and Epidemiology (IMISE), University of Leipzig, Leipzig, Germany

**Keywords:** Center for Epidemiologic Studies Depression Scale, depressive symptoms, mental health, early retirement, pension, population-based study

## Abstract

**Background:**

Transition from employment to retirement is regarded a crucial event. However, there is mixed evidence on associations between retirement and mental health, especially regarding early retirement. In Germany, cases of early retirement due to ill health—particularly, mental ill health—are increasing. Therefore, we investigated the association between early retirement and depressive symptoms, including information on different types of early retirement.

**Methods:**

We analyzed data from 4,808 participants of the population-based LIFE-Adult-Study (age: 40–65 years, 654 retired, 4,154 employed), controlling for sociodemographic information, social network, pre-existing health conditions, and duration of retirement. Depressive symptoms were assessed using the Center for Epidemiologic Studies Depression Scale. Regression analysis using entropy balancing was applied to achieve covariate balance between retired and employed subjects.

**Results:**

We found no overall-differences in depressive symptoms between employed and retired persons (men: b = −.52; p = 0.431; women: b = .05; p = .950). When looking at different types of early retirement, ill-health retirement was linked to increased depressive symptoms in women (b = 4.68, 95% CI = 1.71; 7.65), while voluntary retirement was associated with reduced depressive symptoms in men (b= −1.83, 95% CI = −3.22; −.43) even after controlling for covariates. For women, statutory retirement was linked to lower depressive symptomatology (b = −2.00, 95% CI = −3.99; −.02).

**Conclusion:**

Depressive symptomatology among early retirees depends on reason for retirement: For women, ill-health retirement is linked to higher levels of depressive symptoms. Women who retire early due to ill-health constitute a risk group for depressive symptoms that needs specific attention in the health care and social security system.

## Introduction

Retiring from employment is considered a crucial event, affecting social relationships and roles, daily activities, and possibly various health outcomes (1–3). However, evidence on associations between retirement and mental health remains inconclusive (4–7). This applies particularly to early retirement, i.e., withdrawal from the labor force before reaching the country-specific statutory retirement age. One possible explanation for the mixed results on associations between early retirement and mental health is that most existing studies focus on one specific type of retirement (e.g., retirement due to ill-health or due to layoffs) and/or do not assess different reasons for retirement (2, 8, 9). Comprehensive investigations simultaneously considering mental health in different subtypes of retirees are currently rare, especially in Germany.

Knowledge on the health situation of different types of retirees, however, is crucial e.g., for the design of effective prevention and treatment strategies. Information on mental health of different subgroups of retired people is also valuable for the design of effective policy tools. Many welfare systems currently rely on financial incentives in order to encourage people to stay in the labor force; however, these kinds of incentives might not yield the desired effects if workers retire early involuntarily, e.g., due to health impairments (10–12).

In the face of demographic changes and increased life expectancy, policy makers in several Western countries have implemented different strategies to reduce pathways into early retirement in order to prolong individual careers and increase the share of older employees in the workforce (11, 13, 14). This also applies to Germany, where the statutory retirement age has been increased to currently 65 years and 7 months for people born in or after 1964 and will be raised further until the age of 67 years in the year 2029. Furthermore, replacement rates were successively lowered and subsidies for private pension provisions were introduced (15). Despite these political measures and a noticeable increase in the average retirement age, evidence from cross-national studies indicates that retirement transitions in Germany still occur earlier than in other countries like e.g., England or Japan (16).

Ill-health was repeatedly found to be one of the main factors raising the probability of early retirement (2, 4, 8, 10, 14, 17, 18). Depression, among other mental disorders, constitutes a particularly relevant reason for early retirement (2, 17–19). According to data from the German pension insurance, claims to disability pension due to psychiatric disorders have been increasing continuously during the last years from 20.1% in 1996 to 42.7% of all claims in 2018 (20). Currently, depressive episodes constitute the 3^rd^ most important reason for sick leave days (21). On average, sick leave due to mental ill health lasts 26.1 days. Evidence from Germany revealed that sick leave due to depression is a significant predictor for early retirement (22). This highlights the crucial role of mental health in maintaining work ability and prolonging individual careers. Studies from Scandinavian countries found depression and depressive symptoms to be associated with disability pension due to mental ill-health, but also with retirement due to somatic causes and non-illness-based retirement (17, 18). Depression often occurs as a recurring disease, which is one reason why it can severely disrupt labor force participation (23). Representative data from Germany estimate a share of 60-75% of all cases of depression being recurring depressive disorders (24).

Disability benefits can be granted for those workers having contributed to the statutory pension insurance who have become unable to pursue gainful employment due to ill health or disability. These benefits are only granted if employment capacity cannot be regained through e.g., means of medical rehabilitation and if the applicant is unable to perform any kind of paid work he or she can reasonably follow (25). Receiving a disability pension therefore comes with many preconditions. In 2018, 43% of all applications were denied (20). However, receipt of disability pensions is at least somewhat selective, since not all workers in Germany contribute to the statutory pension insurance; moreover, some people—although maybe suffering from ill-health—might rather look for a less demanding occupation, reduce working hours or leave the working environment through other exit routes, e.g., by becoming a homemaker.

Aside from health reasons, prematurely leaving the work force can occur voluntarily or due to other reasons, e.g., layoffs or corporate reorganization. Voluntariness of the transition from employment to retirement has been found a crucial factor impacting mental health of retirees, with people retiring involuntarily experiencing higher levels of depressive symptoms and other mental health problems than employees retiring voluntarily or because they reached the statutory retirement age (7, 26–28). This is likely because involuntary early retirement severely affects personal retirement plans and comes with financial losses and increased uncertainty (29, 30). Moreover, the skills and experiences of older workers might not easily be transferrable to other occupations, reducing the chance for reemployment (7).

By comparison, people who describe their retirement status as “voluntary” can be expected to report better mental health. As retirement was actively chosen, the transition was likely anticipated and planned, i.e., people deciding to retire voluntarily should be more open to the new experiences and changes that retirement can entail than people who continue to work until the statutory retirement age (26, 31). Several studies investigating the reasons for early retirement found the wish to enjoy life while still being in good health an important factor (32, 33). Moreover, voluntarily leaving the labor force prematurely requires some sort of personal wealth and assets to rely upon. Therefore, it is reasonable to assume that people actively choosing to retire early can draw upon personal resources for retirement. Several studies found voluntary early retirement to be linked to improved mental health and health satisfaction, although the association tended to diminish over time (7, 29, 34).

Although the statutory retirement age in Germany currently amounts to 65 years, certain exceptions apply for employees who entered the labor market early in life and contributed to the statutory pension insurance for at least 45 years, offering the possibility to retire at the age of 63 at full benefit receipt. Special regulations also apply to certain occupational groups, allowing e.g., police officers, soldiers or miners to retire at an earlier age. As outlined above, different reasons for retirement have been found to relate differently to mental health. Therefore, statutory early retirees should be carefully differentiated from those who retired due to specific reasons.

Evidence on possible gender differences concerning the association between mental health and early retirement remains inconclusive, partly because previous studies often exclusively focused on men’s retirement (35). Drawing on traditional societal norms emphasizing the role of employment for men’s identities, it has been suggested that prematurely exiting the labor force might be an especially stressful experience for men, resulting in higher levels of psychological distress than in women who retire early (36). However, given the higher labor force attachment of younger cohorts of women and the changing of gender roles, it has to be questioned whether this assumption still holds true. Possible gender differences might prevail regarding both reasons for retirement and mental health in retirement (28). Previous studies on reasons for retirement found caring duties for a spouse or other family members to be more important for women’s retirement decision than men’s (37, 38). While the overall-rates of retirement due to disability have been decreasing in Germany during the last decades, the share of women receiving a disability pension has increased continuously, partly due to the increased labor force participation of women (39). Lastly, the overall-prevalence of depression is consistently reported higher in women than in men (40). These factors highlight the need for gender-specific information on the association of early retirement and mental health.

Against this background, it can be assumed that different reasons for retirement relate differently to mental health. We therefore hypothesize that **a)** ill-health related retirement is linked to increased depressive symptomatology, while **b)** voluntary early retirement is associated with less depressive symptoms. Involuntary early retirement is assumed to be linked to increased depressive symptomatology **c**). For early retirement due to reaching the statutory retirement age, i.e., where none of these reasons apply, we suspect no association with depressive symptoms **d**). To investigate possible gender differences in the association of early retirement and depressive symptoms, all analyses were conducted separately for men and women **e**).

## Materials and Methods

### Participants

Data were drawn from the LIFE-Adult-Study, a population-based cohort study conducted by the Leipzig Research Center for Civilization Diseases; 10,000 randomly selected citizens of Leipzig, Germany aged between 18 and 79 years completed the baseline examination between 2011 and 2014.

The LIFE-Study aims to investigate the prevalence, genetic predispositions, and modifiable lifestyle factors of major civilization diseases such as cardiovascular diseases, dementia, or depression. The baseline assessment consisted of physical examinations, structured interviews, and questionnaires which were administered to all participants. Pregnancy and insufficient command of the German language were exclusion criteria. For a detailed description of the study aims and concept, please see (41). The study included an age- and sex-stratified random sample of 10,000 community-dwelling German-speaking residents of the city of Leipzig who were randomly drawn from lists provided by the local registry office. These residents were sent an invitation letter, informing them about the aims and design of the study, and a response form. Residents who did not respond were sent a reminder invitation. Non-responders were searched in public phone directories and contacted by phone. For residents who refused to participate, residents of the same age and sex were randomly drawn from the registry office’s lists and invited to participate. The study was approved by the responsible ethics board of the Medical Faculty of the University of Leipzig. All participants provided written informed consent to participate prior to enrolment.

Of the initial sample, we excluded cases younger than 40 and older than 65 years (n = 3,561) in order to exclude cases that had already left the labor market due to old-age retirement or who were too young to be reasonably compared to early retirees; however, there was only one observation younger than 40 years in early retirement. Observations were further excluded if they were neither in employment or retirement (e.g., homemakers, unemployed, on maternal leave; n = 772) or if information was missing on social network (n = 308), net equivalent income (n = 92), job status (n = 10), severe preexisting health conditions (n = 71), type of retirement (n = 5), or depressive symptoms (n = 373). The final sample thus consisted of 4,808 individuals.

### Measures

#### Depressive Symptoms

Depressive symptoms were assessed using the German version of the Center for Epidemiologic Studies Depression Scale [CES-D (42)]. This self-report scale consists of 20 items, assessing symptoms such as depressed mood, hopelessness, or insecurity during the last week, using a 4-point-Likert-scale (0 = never/almost none of the time; 3 = most or all of the time). Possible scores range from 0 to 60 points, with higher values indicating higher levels of current depressive symptomatology. Reference values from comparable population-based samples suggest a cut-off value of ≥ 23 points as an indicator for risk of depression (43).

#### Early Retirement

We investigated observations up to 65 years of age since this represents the legal retirement age in Germany to date. The age of 65 has long served as a benchmark in the German retirement system and is also often chosen for research conducted in other countries as an indicator for “on-time” or statutory retirement, making it feasible to embed our results in a wider international context. Due to certain exceptions in the German pension system (see *Background*), a certain amount of statutory early retirees is likely to be included in the sample, despite the focus on other subtypes of early retirement. This category is to be differentiated from voluntary early retirement: while statutory early retirees worked in their respective jobs until the statutory retirement age, voluntary early retirees exit the labor market prematurely. These early retirees usually rely upon personal assets like e.g., private pension insurance plans in old age or made additional voluntary contributions to the statutory pension insurance during their employment career, allowing for an early withdrawal from the labor force.

In LIFE-Adult, different types of retirement were identified by asking the question “which of the following best describes your situation?” if participants indicated having retired, with the following response categories: retirement due to reaching the statutory retirement age; early retirement due to ill-health; voluntary early retirement; involuntary retirement due to operational circumstances; involuntary retirement due to other reasons. We combined the last two options, resulting in the categories: *employed; statutory retirement; retirement due to ill-health; voluntary early retirement; involuntary early retirement*.

#### Other Covariates

We included gender, age, and marital status (married or living in a partnership *vs.* single, divorced, or widowed) as covariates. Moreover, we included education as measured by the CASMIN-scale [Comparative Analysis of Social Mobility in Industrial Nations (44)], net equivalent income, and job status (high/middle/low) as sociodemographic characteristics. Categorization of jobs was conducted using a scoring algorithm for the measurement of socioeconomic status in epidemiological studies which is based on the International Socio-Economic Index of Occupational Status [ISEI; (45)]. Further, we included information on participants’ social network using the short form of the Lubben Social Network Scale (LSNS-6). The LSNS-6 is a measure widely used for the assessment of social engagement and perceived social support, containing questions like: “how many relatives/friends do you see or hear from at least once a month?; how many relatives/friends do you feel close to such that you could call on them for help?”. Scores range from 0 to 30, with higher values indicating higher levels of social engagement. To control for pre-existing health conditions, we included information on lifetime diagnoses of diabetes, myocardial infarction, or stroke, since these conditions were repeatedly found to increase the propensity of early retirement (46–48). Other conditions reported to be related to early retirement (cancer, arthritis, multiple sclerosis) were not investigated due to high numbers of missing values. If participants described themselves as retired, information on duration of retirement was included in the analyses.

### Statistical Analyses

Comparisons between groups were conducted using Chi²-tests and one-way ANOVA as appropriate. We investigated the association of different types of early retirement and depressive symptoms by means of multivariate linear regression, calculating separate models for men and women, with a p-value < 0.05 indicating significance. Analyses were conducted using Stata 16.0 (SE). A weighting factor provided within the LIFE-data was used to adjust the age- and gender distribution to the German population in 2011 (1^st^ year of baseline assessment). To precisely identify the links between different types of early retirement and depressive symptoms, we first matched retired and employed cases on a set of covariates, namely: age, gender, partnership status, education, job level, net equivalent income, social network size, and pre-existing health conditions. We used entropy balancing to reweigh a matrix of control observations, i.e., employed participants (49). Weighting control observations is performed to achieve maximum comparability between treatment- (i.e., retired) and control observations, even in cases where treatment- and control group differed in covariates before the treatment. This method is more effective than other matching approaches like e.g., propensity score matching since control observations are re-weighted to fulfill pre-specified assumptions like equal means and variances of covariates as in the treatment group (50). Unlike other matching techniques, this algorithm allows to use all available observations in the sample without discarding cases that cannot be matched to a control observation (49). Entropy balancing is a non-parametric approach that takes selection of observations based on time-invariant unobserved variables into account (51). To ensure robustness of our findings, we repeated our analyses using propensity score matching, leading to highly comparable results (not shown).

## Results

### Descriptive Analyses

Among the 4,808 observations, 654 (13.6%) had already left the labor force (men *vs.* women: 39.8/60.2%). This group included 104 (15.9%) cases of early statutory retirement, 196 (30.0%) cases of ill-health retirement, 294 (45.0%) observations who had retired voluntarily and 60 (9.2%) cases of involuntary early retirement. The proportion of women was slightly higher in all subtypes of retirement (61.5, 55.1, 62.6, and 63.3%, respectively). For men, depressive symptoms were lowest in voluntary early retirees (mean = 7.8, SD = 5.7) and highest in the ill-health retirement subgroup (mean = 12.0, SD = 7.4; see **Table 1**). For women, the respective values were lowest in statutory early retirees (mean = 9.2, SD = 5.8) and highest for ill-health retirees (mean = 17.5, SD = 11.2; see **Table 2**). Mean age was significantly lower among employed than among retired subjects (men: mean = 51.0, SD = 6.6; women: mean = 50.6, SD = 6.4). Overall, 81.5% of men and 75.1% of women were married or living with a partner, with the lowest proportion among ill-health retirees, respectively (men: 71.6%, women: 59.3%). Men reporting involuntary early retirement more often belonged to the lowest job-status category (22.7%). Among women, this proportion was highest among ill-health retirees (13.0%). Regarding social networks, ill-health retirees had the lowest mean levels of social support (men: mean = 14.4, SD = 6.0; women: mean = 15.1, SD = 5.0), compared to employed and other retirement subgroups. Observations also differed in terms of education. While 57.5% of early statutory retired men had a high level of education, this proportion was 23.9% among ill-health, 31.8% among voluntary early retirees, and 27.3% among involuntary early retirees (employed: 36.4%). Among women, these educational differences were less pronounced (proportion of women with a high level of education in employment, statutory, ill-health, voluntary, and involuntary early retirement: 35.8, 23.4, 24.1, 28.8, 26.3%, respectively). For men, ill-health retirees had the lowest mean incomes (1,377.1, SD: 724.6), followed by involuntary, voluntary, and statutory retirees and highest values for employed men (mean = 2,236.8, SD = 1,947.9). In the female subsample, mean net equivalent income was lowest among involuntary early retirees (1,174.2, SD = 349.7), followed by ill-health, voluntary, and early statutory retirees and highest values for employed women (1,966.9, SD = 995.4). Regarding pre-existing health conditions, a lifetime diagnosis of stroke was most often found in men retired due to ill-health (10.2%, respectively). Statutory early retired men (30.0%) and ill-health retired women (18.5%) had the highest prevalences for diabetes, while a lifetime diagnosis of myocardial infarction was most frequently found in ill-health retirees (men: 8.0%, women: 5.6%). Average duration of retirement was highest for ill-health retirees (men: mean = 5.6 years, SD = 6.0; women: mean = 7.6, SD = 7.5).

**Table 1:** Sample description of employed and retired respondents, by subgroup (men).

Variable	Employed (unmatched)	Employed (matched)	Statutory early retirement	Ill-health retirement	Voluntary retirement	Involuntary retirement	F/chi² (unmatched)	P-value (unmatched)	F/chi² (matched)	P-value (matched)
Age, mean (SD)	51.0 (6.6)	62.2 (2.8)	64.4 (2.2)	59.2 (6.1)	63.9 (1.3)	63.7 (2.2)	189.28	P<0.001	129.20	P<0.001
Partnership %	81.0	89.8	85.0	71.6	94.5	90.1	20.04	P<0.001	6.72	P<0.001
Job status low %	9.2	17.9	2.5	19.3	19.1	22.7	30.33	P<0.001	1.13	P = 0.339
Job status middle %	83.5	76.2	90.0	78.4	76.4	77.3				
Job status high %	7.4	5.9	7.5	2.3	4.5	0.0				
Education low %	3.2	4.4	0.0.	4.5	6.4	4.5	18.80	P = 0.016	1.93	P = 0.053
Education middle %	60.4	59.0	42.5	71.6	61.8	68.2				
Education high %	36.4	36.7	57.5	23.9	31.8	27.3				
Net equivalent income, mean (SD)	2,236.8 (1,947.9)	1,565.9 (570.5)	1,642.5 (593.9)	1,377.1 (724.6)	1,435.1 (484.7)	1,424.4 (582.6)	10.69	P<0.001	11.67	P<0.001
Social resources (LSNS-6-score), mean (SD)	17.0 (5.3)	16.1 (5.7)	14.7 (5.9)	14.4 (6.0)	16.8 (6.1)	18.1 (4.8)	6.85	P<0.001	14.11	P<0.001
Stroke %	0.6	1.7	2.5	10.2	1.8	0.0	71.74	P<0.001	4.24	P = 0.002
Diabetes %	5.6	14.9	30.0	23.9	10.0	13.6	77.66	P<0.001	2.82	P = 0.0255
Myocardial infarction %	1.2	6.9	7.5	8.0	5.5	0.0	68.15	P<0.001	0.44	P = 0.7625
Pension duration, years			1.4 (1.7)	5.6 (6.0)	2.1 (2.1)	3.1 (2.4)	38.38	P<0.001	38.38	P<0.001
Depressive symptoms (CES-D-score), mean (SD)	9.1 (5.5)	9.7 (5.1)	9.7 (5.1)	12.0 (7.4)	7.8 (5.7)	9.6 (4.7)	7.52	P<0.001	27.93	P<0.001
n	1,894		40	88	110	22				

n = 2,154; table presents results for unmatched and matched controls (employed respondents); age reported in years; education assessed according to CASMIN (Comparative Analysis of Social Mobility in Industrial Nations)-classification categories low, middle, and high; social resources assessed by the Lubben Social Network Scale (LSNS-6); CES-D, Center for Epidemiologic Studies Depression Scale; stroke, diabetes, and myocardial infarction: lifetime diagnoses; p-values based on Chi^2^-tests and one-way ANOVA, as appropriate.

**Table 2:** Sample description of employed and retired respondents, by subgroup (women).

Variable	Employed (unmatched)	Employed (matched)	Statutory early retirement	Ill-health retirement	Voluntary retirement	Involuntary retirement	F/chi² (unmatched)	P-value (unmatched)	F/chi² (matched)	P-value (matched)
Age, mean (SD)	50.6 (6.4)	62.1 (2.9)	64.2 (1.7)	58.2 (6.2)	63.2 (1.8)	63.1 (3.5)	302.72	P<0.001	163.55	P<0.001
Partnership %	75.0	72.2	82.8	59.3	81.0	81.6	20.77	P<0.001	4.05	P = 0.005
Job status low %	4.8	8.5	12.5	13.0	6.5	5.3	28.79	P<0.001	1.20	P = 0.294
Job status middle %	87.1	88.8	79.7	83.3	90.8	89.5				
Job status high %	8.1	2.7	7.8	3.7	2.7	5.3				
Education low %	2.1	3.7	0.0	4.6	4.3	2.6	20.96	P = 0.007	0.54	P = 0.811
Education middle %	62.1	72.5	76.6	71.3	66.8	71.1				
Education high %	35.8	23.8	23.4	24.1	28.8	26.3				
Net equivalent income, mean (SD)	1,966.9 (995.4)	1,316.2 (544.3)	1,546.2 (717.3)	1,260.7 (520.3)	1,466.2 (482.4)	1,174.2 (349.7)	32.75	P<0.001	21.80	P<0.001
Social resources (LSNS-6-score), mean (SD)	17.3 (5.0)	15.9 (5.2)	17.3 (4.5)	15.1 (5.0)	16.5 (4.4)	15.3 (4.0)	7.24	P<0.001	9.23	P<0.001
Stroke %	0.7	6.2	1.6	10.2	1.6.	5.3	84.28	P<0.001	1.30	P = 0.272
Diabetes %	3.6	16.0	10.9	18.5	12.5	13.2	80.96	P<0.001	0.438	P = 0.688
Myocardial infarction %	0.3	1.8	0.0	5.6	1.1	2.6	51.17	P<0.001	1.28	P = 0.280
Pension duration, years			1.5 (1.8)	7.6 (7.6)	2.1 (1.8)	3.6 (2.9)	627.17	P<0.001	627.17	P<0.001
Depressive symptoms (CES-D-score), mean (SD)	10.8 (7.4)	13.1 (7.1)	9.2 (5.8)	17.5 (11.2)	11.0 (5.3)	11.4 (7.7)	21.83	P<0.001	59.67	P<0.001
n	2,260		64	108	184	38				

n = 2,654; table presents results for unmatched and matched controls (employed respondents); age reported in years; education assessed according to CASMIN (Comparative Analysis of Social Mobility in Industrial Nations)-classification categories low, middle, and high; social resources assessed by the Lubben Social Network Scale (LSNS-6); CES-D, Center for Epidemiologic Studies Depression Scale; stroke, diabetes, and myocardial infarction: lifetime diagnoses; p-values based on Chi^2^-tests and one-way ANOVA, as appropriate.

Descriptive sample statistics are provided in **Tables 1**, **2** for men and women, respectively. Results are presented for unmatched, i.e., before entropy balancing, and matched controls, the last four columns reporting information on significance of differences between early retirement subtypes and unmatched/matched control observations, respectively.

### Multivariate Analyses

**Tables 3**, **4** report the results of multivariate linear regression models, stratified by gender. The results of a model comparing employed and retired observations without differentiating for early retirement reasons are shown in **model 1.** No association between retirement and depressive symptoms was detected in either men or women (men: b = −.52, p = 0.431; women: b = .05, p = 0.950). Higher age and a larger social network were linked to lower depressive symptoms in both genders, whereas living in a partnership was associated with lower depressive symptoms only in women (b = −3.37; 95% CI: −5.33, −1.40). Women reporting a lifetime diagnosis of diabetes had increased depressive symptoms. The analyses did not reveal a link between a lifetime-diagnosis of myocardial infarction or stroke and depressive symptomatology. For both men and women, no association between sociodemographic factors (education, net equivalent income, job level) and depressive symptoms could be demonstrated.

**Table 3:** Multivariate linear regression, association of depressive symptomatology (CES-D), and early retirement, men.

	Men (n = 2,154)							
	Model 1				Model 2			
	Coeff.	95% CI		P-Value	Coeff.	95% CI		P-value
Employed	Ref.				Ref.			
Retired	−.52	−1.83	.78	0.431				
Statutory early retirement					.20	−1.66	2.06	0.831
Ill-health retirement					1.34	−.90	3.58	0.240
Voluntary retirement					−**1.83**	−3.22	−.43	0.010
Involuntary retirement					.28	−1.80	2.36	0.795
Age	−**.17**	−.33	−.01	0.034	−.10	−.25	.05	0.187
Partnership (Ref.: single)	.40			0.671	.69	−1.10	2.48	0.447
Job status low	Ref.				Ref.			
Job status middle	−.57	−2.10	.96	0.463	−.78	−2.26	.71	0.306
Job status high	2.48	−1.04	6.01	0.167	2.41	−1.10	5.92	0.178
Education low	Ref.				Ref.			
Education middle	−.56	−2.29	1.17	0.523	−.60	−2.30	1.11	0.491
Education high	−1.97	−3.99	.06	0.057	−1.99	−4.01	.03	0.053
Net equivalent income	−.00	−.00	.00	0.154	−.00	−.01	.00	0.123
Social resources	−**.28**	−.38	−.19	0.000	−**.27**	−.37	−.17	0.000
Stroke	.21	−5.43	5.85	0.941	−.27	−5.90	5.36	0.926
Diabetes	−.15	−1.39	1.09	0.810	−.55	−1.75	.66	0.376
Myocardial infarction	1.03	−1.31	3.38	0.387	1.09	−1.24	3.41	0.361
Pension duration, years	.12	−.13	.37	0.359	.04	−.23	.31	0.768
								
R²	0.1510				0.1718			

Coeff., coefficient; CI, confidence interval; education assessed according to CASMIN (Comparative Analysis of Social Mobility in Industrial Nations)-classification categories low, middle, and high; social resources assessed by the Lubben Social Network Scale (LSNS-6); CES-D, Center for Epidemiologic Studies Depression Scale; stroke, diabetes, myocardial infarction: lifetime diagnoses; significant associations presented in bold type.

**Table 4:** Multivariate linear regression, association of depressive symptomatology (CES-D) and early retirement, women.

	Women (n = 2,654)								
	Model 1					Model 2			
	Coeff.	95% CI		P-value		Coeff.		95% CI	P-value
Employed	Ref.					Ref.			
Retired	.05	−1.39	1.48	0.950					
Statutory early retirement						**−2.00**	−3.99	−.02	0.047
Ill−health retirement						**4.68**	1.71	7.65	0.002
Voluntary retirement						−.65	−2.00	.69	0.340
Involuntary retirement						−.66	−3.49	2.16	0.645
Age	**−.32**	−.51	−.12	0.002		−.15	−.36	.07	0.179
Partnership (Ref.: single)	**−3.37**	−5.33	−1.40	0.001		**−3.07**	−4.95	−1.19	0.001
Job status low	Ref.					Ref.			
Job status middle	.54	−1.16	2.24	0.532		.42	−1.34	2.19	0.638
Job status high	2.45	−1.27	6.18	0.197		2.35	−1.19	5.89	0.193
Education low	Ref.					Ref.			
Education middle	−2.26	−5.45	.94	0.166		−2.53	−6.01	.95	0.154
Education high	−3.05	−6.11	.66	0.115		−3.13	−6.75	.50	0.091
Net equivalent income	−.00	−.00	.00	0.077		−.00	−0.00	.00	0.159
Social resources	**−.34**	−.47	−.20	0.000		**−.31**	−.45	−.18	0.000
Stroke	2.95	−2.48	8.38	0.286		2.79	−2.56	8.14	0.306
Diabetes	**2.58**	.59	4.57	0.011		**2.18**	.25	4.11	0.027
Myocardial infarction	2.59	−.34	5.52	0.084		2.20	−.73	5.13	0.142
Pension duration, years	−.02	−.17	.16	0.978		−18	−.38	.02	0.071
								
R²	0.1697					0.2039			

Coeff., coefficient; CI, confidence interval; education assessed according to CASMIN (Comparative Analysis of Social Mobility in Industrial Nations)-classification categories low, middle, and high; social resources assessed by the Lubben Social Network Scale (LSNS-6); CES-D, Center for Epidemiologic Studies Depression Scale; stroke, diabetes, myocardial infarction: lifetime diagnoses; significant associations presented in bold type.

**Model 2** presents the results of a multivariate regression model differentiating between reasons for early retirement, i.e., statutory retirement, retirement due to ill-health, voluntary, and involuntary early retirement (reference group: employment). Men who retired voluntarily showed lower depressive symptoms (b = −1.83, 95% CI = −3.22; −.43), while no significant differences were detected for other groups of retirees in respect to depressive symptoms. A larger social network was linked to lower depressive symptoms. Results in the female subsample followed a different pattern. While women in statutory retirement showed lower levels of depressive symptoms (b = −2.00; 95% CI = −3.99; −.02), women reporting retirement due to ill-health had increased depressive symptoms (b = 4.68; 95% CI = 1.71; 7.65). Living in a partnership and a larger social network were associated with lower depressive symptomatology in women, while a lifetime diagnosis of diabetes was linked to increased depressive symptoms. No other associations between covariates and depressive symptoms were revealed in either men or women.

## Discussion

Overall, no differences between early retirees and men and women in employment regarding depressive symptoms were detected. However, on further investigation of different reasons for retirement, a more complex picture emerged. Possible interpretations of our findings are discussed below.

*Voluntary early retirement* was linked to lower depressive symptoms in men, but not in women. The negative association between voluntary early retirement and depressive symptoms in men might point toward the importance of voluntariness, i.e., whether a true choice to retire was present. This finding is supported by earlier studies, stating that voluntary retirement is protective for mental health, compared to other kinds of retirement (7, 26, 29, 34). For men retiring voluntarily, the expected benefits from retirement might outweigh the advantages of employment. Considering the higher proportion of women reporting voluntary early retirement, the non-significant association in women seems surprising at first. However, the female sample of voluntary early retirees might be more diverse than captured by our assessments. As e.g., ill-health retirement benefits come with many preconditions (see *Background*), certain women might not apply for the respective benefits or leave the labor market early for other reasons, e.g., to become a homemaker, and describe their decision as voluntary. This interpretation is in line with previous studies suggesting that different self-definitions of employment status between older men and women contribute to gender differences in the association of retirement and depressive symptoms (9). Moreover, women still engage in care work more often than men even at higher ages and in retirement (52). Findings from German panel data and the Australian HILDA-Survey, reporting that caregiving duties have a stronger influence on women’s decision to retire than on men’s (37, 38), underscore this interpretation. This could have contributed to the non-significant association of voluntary retirement and depressive symptoms in women.

Early retirement due to *ill-health*, on the other hand, was linked to higher levels of depressive symptoms only in women. One possible explanation for this finding is health selection into ill-health retirement, since women experience higher overall-levels of depression than men in the general population. Furthermore, the causes for disability retirement might be gender-specific: Evidence from German pension insurance data revealed that mental disorders accounted for 36.3% of men’s ill-health retirement cases, with 6.9% of these cases due to addictions; in women, mental disorders made up 48.7% of all disability pensions, with only 1.9% of these cases related to addictions. Disability retirement due to diseases of the circulatory system, on the other hand, were more prominent in men (13.6% of all cases) than in women [5.6%; (53)].

Contrary to our hypothesis, no association between *involuntary early retirement* and depressive symptoms was detected in our sample. However, there is evidence from studies on unemployment and mental health, suggesting that unemployed people experience lower levels of mental health problems if the reason for job loss is perceived as exogenous, e.g., in cases of plant closures or layoffs [for a review, please see (54)]. If the reason for losing one’s job is not perceived as individual failure but rather as a result of a company’s/employer’s decision, involuntary early retirement might show no association with depressive symptoms. Moreover, the probability of health selection is rather small in cases of layoffs. However, our sample only contained a small number of involuntary early retirees, therefore, interpretation of these results should be made with caution.

S*tatutory early retirement* was linked to lower depressive symptomatology only in women, suggesting that retirement due to reaching the statutory retirement age is beneficial for women’s mental health. For these women, retirement might be perceived as a relief and a possibility to engage in other roles or enjoy a greater amount of leisure time. This corroborates findings from the US-American Health and Retirement Survey, reporting improved mental health in women following retirement (7). On the other hand, no association of statutory retirement and depressive symptoms was found in men. However, our study investigated men and women up to the age of 65, i.e., where statutory retirement was a rather recent event. Therefore, we cannot conclude whether these gender differences in mental health among statutory early retirees persist throughout retirement or whether an association of depressive symptoms and statutory retirement might emerge at a later time-point for men.

Social networks, as captured by the number of family and non-family contacts, were smaller in all subgroups of early retirees, compared to those in employment, a finding that is consistent with previous studies (27, 55). Larger social networks were linked to lower depressive symptoms both in men and women, corroborating previous studies reporting a protective effect for social networks and support (27). Retirement can bring about more time for leisure activities that can be enjoyed with friends, but our finding could also point toward the potentially buffering effect of friendships and social resources in times of meaningful transitions and change, e.g., the transition to retirement. A partnership, however, was only protective for female retirees, highlighting the importance of marriage and partnership for the mental health of women. This finding underscores previous studies reporting greater importance of marriage/partnership for female retiree’s mental health (35, 56, 57).

The non-significant impact of income on the association of early retirement and depressive symptoms detected in our sample seems surprising at first sight. However, this factor draws upon information on net equivalent income at the time of the interview which is only one indicator of peoples’ economic situation; another indicator could be e.g., net assets, which give a hint about savings people accumulated over their lifetime and can rely on when exiting the workforce (58). In the face of increasing life expectancy and, therefore, longer periods of retirement, being able to rely on personal assets for retirement is crucial (23). Certain longitudinal studies found associations between changes in income after retirement and mental health (59). Unfortunately, however, this information was not available since our study relied on cross-sectional data.

In line with findings from other investigations (59, 60), no association between level of education and depressive symptoms was found in our sample. This implies that the association of different types of retirement and depressive symptoms is rather independent of educational attainments. It has to be pointed out, however, that average levels of education were rather high in our sample and only a small fraction of observations belonged to the lowest education category.

Similar results were found for job status, with no impact on the association between early retirement and depressive symptoms in either men or women. However, job-level is a rather general descriptor of (previous) employment; possibly, there might be differences between certain occupations or job characteristics which provide different levels of resources for mental health. Further investigations on the mental health of retirees from different occupations might provide useful insights. Chandola et al. (61), using data from the Whitehall II study, found different trajectories of mental health between employment grades over time (improved mental health for retirees from high-grade jobs, but no similar trend for low-grade jobs), but no baseline differences between the respective groups. According to these findings, possible links between socioeconomic position and depressive symptoms might only emerge over a longer time span following retirement.

### Strengths and Limitations

A major strength of our study lies in the use of a large, population-based sample, which might likely be less selective than samples from previous studies. Furthermore, our data included information on distinct reasons for early retirement, i.e., statutory, voluntary, involuntary, or ill-health-related retirement, allowing for nuanced statements about the links between retirement and depressive symptoms. Due to the extensive set of covariates included in the LIFE-Adult-Study, we were able to control for a number of important factors that possibly impact the relationship between retirement and depressive symptoms. We addressed possible heterogeneity between employed and retired subjects by using advanced techniques of covariate balancing, namely: entropy balancing, which should make our findings robust against selection bias, a problem shared by many comparable studies in the field. Further, we were able to provide information on the association of retirement status and depressiveness for both genders. Many previous studies primarily focused on men’s retirement and mental health, often assuming a stronger labor market attachment in men or presuming single-earner households. However, since labor force participation of women has increased continuously in the last decades and gender roles are not fixed but changing over time, providing information on women’s mental health in retirement is crucial for a comprehensive understanding of the association between retirement and depressiveness.

Certain limitations need to be kept in mind when interpreting our findings. A sizeable proportion of observations had to be discarded due to missing values in the variables of analysis. Although we controlled for several possible confounders in our analyses, other factors possibly impacting the link between early retirement and depressive symptoms were not available in the LIFE-Adult assessment. This applies e.g., to the health- and employment status of spouses/partners or care obligations for a spouse or other family members, factors found to have a significant impact especially on women’s health in retirement (36, 38). Moreover, there were no information available on which diagnoses lead to ill-health retirement, a factor that could have shed light on the observed gender differences in the ill-health retirement subsample. Bearing in mind that depression diagnoses increase the risk of disability pensions, it is likely that a certain amount of ill-health retirees in our sample left the labor market due to depression. Therefore, health selection is likely to have contributed to our findings.

Since our study relied on cross-sectional data, we cannot draw conclusions as to a causal relationship between depressive symptoms and early retirement status or about the course of depressive symptoms post-retirement. Several studies have pointed out that the relationship between retirement and depressive symptoms is reciprocal, i.e., depressive symptoms increase the likelihood of retirement intentions (62) and actual retirement transitions (18, 63, 64), but retirement can also increase depressive symptoms (9) or psychological distress (36). Since the LIFE-Adult-Study has a longitudinal cohort design with an extensive follow-up-period, longitudinal investigations of the association between early retirement and depressive symptoms will be feasible in the near future.

Our study mainly included retirees who only recently transitioned to retirement. However, the link between retirement status and depressive symptoms might change over time as people adjust to retirement. Engagement in leisure activities and availability of alternative social relationships (apart from colleagues and co-workers) have been found important indicators of retirement adjustment (30, 65–67), and previous studies found an increase in social or physical leisure activities in retirement to be associated with decreased depressive symptoms (68). On the other hand, a recent meta-analysis reported a significant reduction of social engagement and integration in early retirees over time, which was not compensated by non-work related contacts (65). Therefore, longitudinal analyses investigating the association of social resources and mental health in early retirees seem worthwhile.

Previous studies investigating the impact of work-related factors on the decision to retire early stressed the importance of factors like job strain or high demands at work; high levels of job strain, increasing mental demands at work and conflicts in the workplace were found to raise the likelihood of early retirement (14, 69). The Danish SeniorWorkingLife study identified further work-related factors impacting older workers’ retirement intentions: for lower-status and physically demanding jobs, poor physical health, and inability to perform their current job increased the intention to retire, while for higher-status and predominantly sedentary jobs, more personal freedom and a preference for leisure activities were important driving factors (66). On the other hand, previous investigations found evidence that positive job attributes like meaningful tasks and high levels of control are able to increase employees’ intention to prolong their working life post retirement age (70). Unfortunately, no such information was available in our data. However, the first follow-up assessment of the LIFE-Adult-Study which is currently undertaken includes several instruments addressing work-related psychosocial factors, making respective investigations possible in the near future.

## Conclusion

We were able to provide detailed insights on the association between early retirement and depressive symptoms, using data from a large population-based study. Although the German welfare system has been subject to major changes in the last decades, i.e., a departure from various possibilities for premature labor market exit toward an active ageing strategy and the promotion of prolonged employment careers, early retirement is still a common phenomenon. Hence, detailed information on the reasons for early retirement and the mental health situation of older workers and early retirees is urgently needed.

Our findings provide evidence that the group of early retired men and women is quite heterogeneous, whereby the reason for early labor market exit is critical for the association with depressive symptoms. Retirement due to ill-health was linked to higher levels of depressive symptoms for women, suggesting that this group is at an increased risk for depressiveness. This finding is of particular importance since many previous studies primarily focused on men’s retirement. Further studies investigating different reasons for early retirement in both genders are highly desirable, since younger cohorts of women might likely show a stronger labor force attachment than in previous studies with older populations. Moreover, since care work and other non-work responsibilities still tend to be unequally distributed between men and women, future investigations should further account for the individual experience of and social roles fulfilled in early retirement and possible links to depressiveness in both women and men.

Regarding possible prevention strategies, a strong focus should be placed on maintaining mental health and preventing depressive symptoms in the workforce in order to prevent cases of ill-health related retirement. Data from German health- and pension insurances reveal that depression increases the risk for retirement due to a depression diagnosis, but also other cases of early withdrawal from the labor force. Future studies investigating specific stressors in the workplace that might be linked to depressiveness could provide useful insights on possible approaches for occupational health managers and political actors. Furthermore, studies investigating subjective reasons for retirement in different occupational groups could shed further light on the factors driving retirement transitions and possible relationships with mental health. This knowledge could help inform the design of tailored workplace interventions and policy strategies to prolong working lives and preserve mental health in different groups of older workers.

Other approaches should address the preservation of mental health in those women who already retired due to health reasons in order to avoid depressive symptoms. Our findings revealed evidence for the crucial role of social networks, therefore, prevention strategies against depressive symptoms should include encouragement for maintaining social contacts and relationships in retirement. Future studies investigating social roles and activities pursued in retirement could provide valuable insights into this complex association. Regarding possible back-to-work-strategies for this subgroup, job placement programs supporting workers in their re-entry into the competitive labor market, e.g., by the means of personal job coaches, have been found an effective approach for workers suffering from depression (71, 72). However, further research is needed to assess the feasibility of such programs for ill-health retirees.

## Data Availability Statement

The data analyzed in this study is subject to the following licenses/restrictions: the dataset analyzed during the current study is available from the corresponding author upon request. Requests to access these datasets should be directed to Andrea.Zuelke@medizin.uni-leipzig.de.

## Ethics Statement

The studies involving human participants were reviewed and approved by Ethics board of the Medical Faculty of the University of Leipzig. The patients/participants provided their written informed consent to participate in this study.

## Author Contributions

AZ conducted the statistical analyses, interpreted the data and drafted the manuscript. SR supported in interpreting the data and drafting the manuscript. MS, AW, AH, HG, ChE, SiZ, SaZ, and CoE revised the manuscript for intellectual content, read and approved the final version of the manuscript. ML and AV conceptualized and designed the study, revised the manuscript for intellectual content, read and approved the final version of the manuscript. SRH conceptualized and designed the study, supervised the drafting of the manuscript, supported in interpreting the data, revised the manuscript for intellectual content, read and approved the final version of the manuscript.

## Funding

This publication is supported by LIFE – Leipzig Research Center for Civilization Diseases, University of Leipzig. LIFE is funded by means of the European Union, by the European Regional Development Fund (ERDF) and by means of the Free State of Saxony within the framework of the excellence initiative. This project was funded by means of the European Social Fund and the Free State of Saxony.

## Conflict of Interest

The authors declare that the research was conducted in the absence of any commercial or financial relationships that could be construed as a potential conflict of interest.

## Abbreviations

ANOVA, analysis of variance; CASMIN, Comparative Analysis of Social Mobility in Industrial Nations; CES-D, Center for Epidemiologic Studies Depression Scale; CI, confidence interval; LSNS-6, Lubben Social Network Scale; SD, standard deviation.
